# Private Medical Instruction

**Published:** 1859-03

**Authors:** 


*Private Medical Instruction.*—By reference to the advertising sheet, it will be seen that Prof. Austin Flint and the editor, propose to form a class during the coming summer, for private instruction in branches which cannot be so elaborately taught in the regular course. This will commence on the first of May and continue six months, embracing practical lessons in all departments of physical exploration and clinical medicine, at the Buffalo Hospital of the Sisters of Charity, with a course of microscopic anatomy and the application of the microscope to the various departments of medicine. Facilities for this kind of instruction are abundant in Buffalo, and it is hoped that those pursuiug this course will be enabled to learn, *practically*, the important applications of physical exploration to the diagnosis of disease, and the use of microscope, which has of late become so important to the practitioner.

